# Fetal Hemoglobin and Tissue Oxygenation Measured With Near-Infrared Spectroscopy—A Systematic Qualitative Review

**DOI:** 10.3389/fped.2021.710465

**Published:** 2021-08-13

**Authors:** Ena Pritišanac, Berndt Urlesberger, Bernhard Schwaberger, Gerhard Pichler

**Affiliations:** ^1^Division of Neonatology, Department of Pediatrics, Medical University, Graz, Austria; ^2^Research Unit for Neonatal Micro- and Macrocirculation, Medical University of Graz, Graz, Austria

**Keywords:** fetal hemoglobin, newborn, near infrared spectroscopy, cerebral tissue oxygenation, fractional oxygen extraction

## Abstract

Fetal hemoglobin (HbF) is a principal oxygen carrier in the blood of preterm and term neonates. Compared to adult hemoglobin, it has a significantly higher affinity for oxygen and its oxyhemoglobin dissociation curve (ODC) is left-shifted accordingly. Tissue oxygenation measured with near-infrared spectroscopy (NIRS) during neonatal intensive care is directly affected by hemoglobin concentration. We performed a systematic qualitative review regarding the impact of HbF on tissue oxygenation monitoring by NIRS. The PubMed/Medline, EMBASE, Cochrane library and CINAHL databases were searched from inception to May 2021 for studies relating to HbF and NIRS in preterm and term neonates in the first days and weeks after birth. Out of 1,429 eligible records, four observational studies were included. Three studies found no effect of HbF on cerebral tissue oxygenation. One peripheral NIRS study found a positive correlation between HbF and peripheral fractional oxygen extraction (FOE). Currently available limited data suggest that FHbF could affect peripheral muscle FOE, but seems not to affect cerebral oxygenation in preterm neonates. More studies are needed to draw a final conclusion on this matter, especially concerning the oxygenation changes driven by adult RBC transfusions.

## Introduction

**Figure 1:**
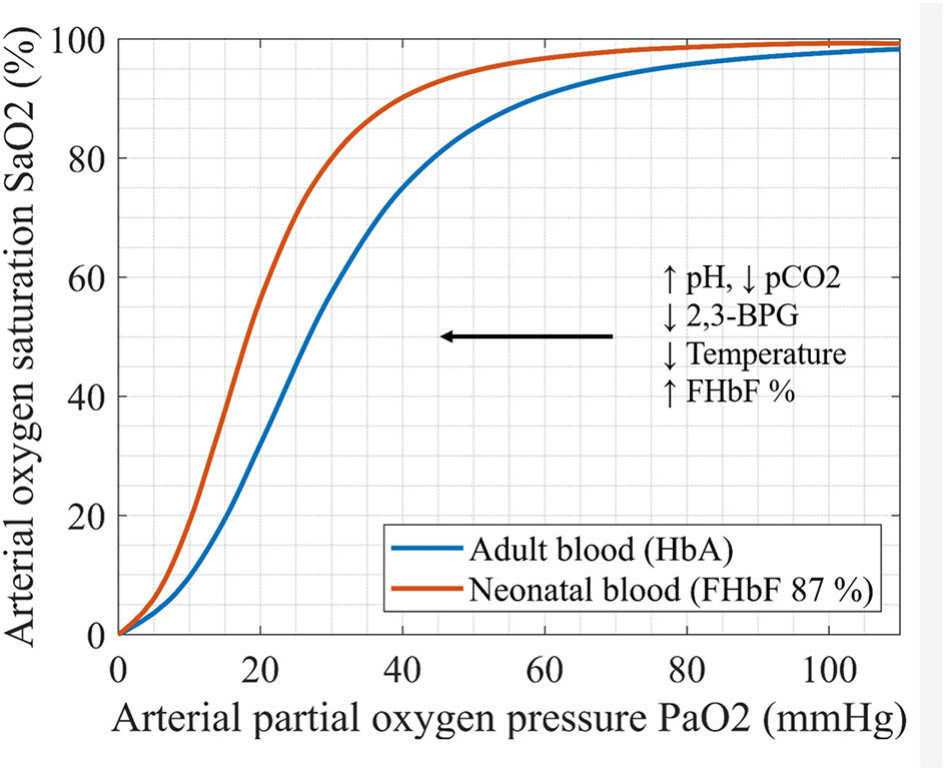
Oxyhemoglobin dissociation curves (ODC) of adult and neonatal blood sample: ODC reflects the relationship between paO2 and SaO2. The factors that change the oxygen affinity for hemoglobin are illustrated (pCO2-partial pressure of carbon dioxid, 2,3-BPG- biphosphoglycerate, FHbF-fraction of fetal hemoglobin).

The oxygen carrying capacity of blood depends primarily on the hemoglobin molecule. Fetal hemoglobin (HbF) is a principal oxygen carrier in both preterm and term neonates. Compared to adult hemoglobin (HbA), HbF exhibits a higher affinity for oxygen and a decreased affinity for 2,3-biphosphoglycerate (2,3-BPG). This results in a left-shifted oxyhemoglobin dissociation curve (ODC) of HbF relative to HbA. The leftward shift in the ODC ensures an oxygen uptake at a lower partial oxygen pressure (pO2) for fetus *in utero*, as well as a lower oxygen extraction at capillary beds in peripheral tissues (1–5) (Figure 1).

The amount of HbF in blood is often expressed as a percentage of total hemoglobin or fraction of fetal hemoglobin (FHbF). A set of evolutionary conserved genes regulates the postnatal expression of HbF and genetic switch to HbA production. This process is not affected by the birth event itself and evolves gradually over a period of at least 6 months (2). Nevertheless, a study in more than 150,000 neonates of different gestational ages (22–42 weeks of gestation), reported a respectable variation in FHbF, especially in the term neonates at birth with a mean FHbF of 82% (min–max 5–100%). This suggests that FHbF at birth, and its eventual decline is an individual characteristic of each term and preterm neonate (6). Higher FHbF were observed in neonates exposed to risk factors for maternal or fetal hypoxia (7). Furthermore, in very low birth weight neonates, higher FHbF were related to the lower incidence of retinopathy of prematurity (ROP), suggesting that HbF could be a protective factor for oxygen-related tissue injury in preterm neonates (8).

A continuous non-invasive oxygenation monitoring is of great interest during postnatal resuscitation as well as during neonatal intensive care (9). Pulse-oximetry is the most common oxygenation monitoring method, based on detecting changes in the absorption of oxygenated and deoxygenated blood hemoglobin at two wavelengths: 660 nm (red) and 940 nm (infrared) (10). Nonetheless, arterial saturation measured by pulse-oximetry (SpO2) provides only the information about the saturation of arterial blood without giving any insight into oxygen consumption. Moreover, SpO2 values show a respectable bias, when compared to direct measurements of arterial blood saturation (SaO2) in neonates, which can result in an undetected hypoxia (11–13).

Tissue oxygenation monitoring by near-infrared spectroscopy (NIRS) enables the assessment of oxygen delivery to the end organs, most commonly to the brain, peripheral muscle or kidney/flank. The method is based on the absorption changes of oxygenated and deoxygenated blood hemoglobin in near-infrared part of the spectrum (700–1,000 nm) and reflects a mixed tissue saturation (14, 15).

NIRS measurements in neonates are, therefore, a matter of increasing interest in neonatal intensive care, especially in assessing cerebral tissue oxygenation in the first minutes after birth (16, 17). On the one hand insufficient oxygen supply can cause hypoxic tissue damage and on the other hand an excess in oxygen supply increases the risk of oxidative stress (18). NIRS monitoring of cerebral tissue oxygenation in combination with treatment guidelines reduces the burden of both cerebral hypoxia and hyperoxia (19).

There are several factors, which affect cerebral tissue oxygenation and oxygen extraction in neonates such as gestational age, postnatal age, brain injury, total hemoglobin and blood transfusions (20–22).

Simultaneous measurements of tissue oxygenation and SpO2 combined with a venous occlusion (for peripheral measurements) enable the calculation of fractional oxygen extraction (FOE), i.e., the amount of oxygen that is extracted from blood to a tissue (23). Venous occlusion causes an increase in blood volume by interruption of venous (out) flow, whereas arterial (in) flow remains unaffected. Thus, the measured changes in oxygenated, reduced and total hemoglobin during venous occlusion are caused only by the arterial inflow and oxygen consumption of tissue (24).

Based on the physiological characteristics of HbF, in particular its left-shifted ODC, the individual differences in FHbF and therefore in hemoglobin affinity for oxygen, are expected to affect the measured cerebral and peripheral oxygenation and oxygen extraction. Since NIRS measurements mainly reflect the venous department (arterial:venous contribution = 25:75) (25) higher FHbF should lead to lower oxygen extraction, since the unfolding of oxygen from the HbF molecule requires lower partial oxygen pressures (26). After a transfusion of adult red blood cells (RBCs), on the other hand, the shift to the right in ODC (Figure 1) should enhance oxygen unfolding and lead to an increase in FOE.

The aim of this review is to summarize the studies, which investigated whether the individual differences in FHbF affect cerebral and peripheral tissue oxygenation or FOE measured by NIRS.

## Methods

### Search Strategy

Articles were identified using the stepwise approach according to the Preferred Reporting Items for Systematic Reviews and Meta-Analyses (PRISMA) statement (27). We performed a systematic search of PubMed/Medline, EMBASE, Cochrane library and CINAHL databases for articles published between inception of the databases and May 2021 that addressed HbF and tissue oxygenation monitoring by NIRS in term and preterm neonates. We have applied a search restriction to human studies and to the publications in English. Search terms included: newborn, neonate, preterm, term, infant, HbF, hemoglobin F, fetal hemoglobin, near-infrared spectroscopy, NIRS, cerebral tissue oxygenation, peripheral tissue oxygenation, fractional oxygen extraction (see Supplement 1). Studies on fetal hemoglobin in terms of sickle cell anemia and thalassaemia were excluded. Additional published reports were identified through a manual search of references in retrieved articles and in review articles.

### Study Selection Criteria

The two authors (E.P., G.P.) evaluated the retrieved articles independently by reviewing the titles and abstracts. The full text was analyzed if there was an uncertainty regarding eligibility for the inclusion. The two reviewers independently selected relevant abstracts, critically appraised the full texts of the selected articles, and assessed the methodological quality of the studies. Data were analyzed qualitatively. Extracted data included the characterization of study type, patient characteristics, methods, devices and results.

## Results

**Figure 2:**
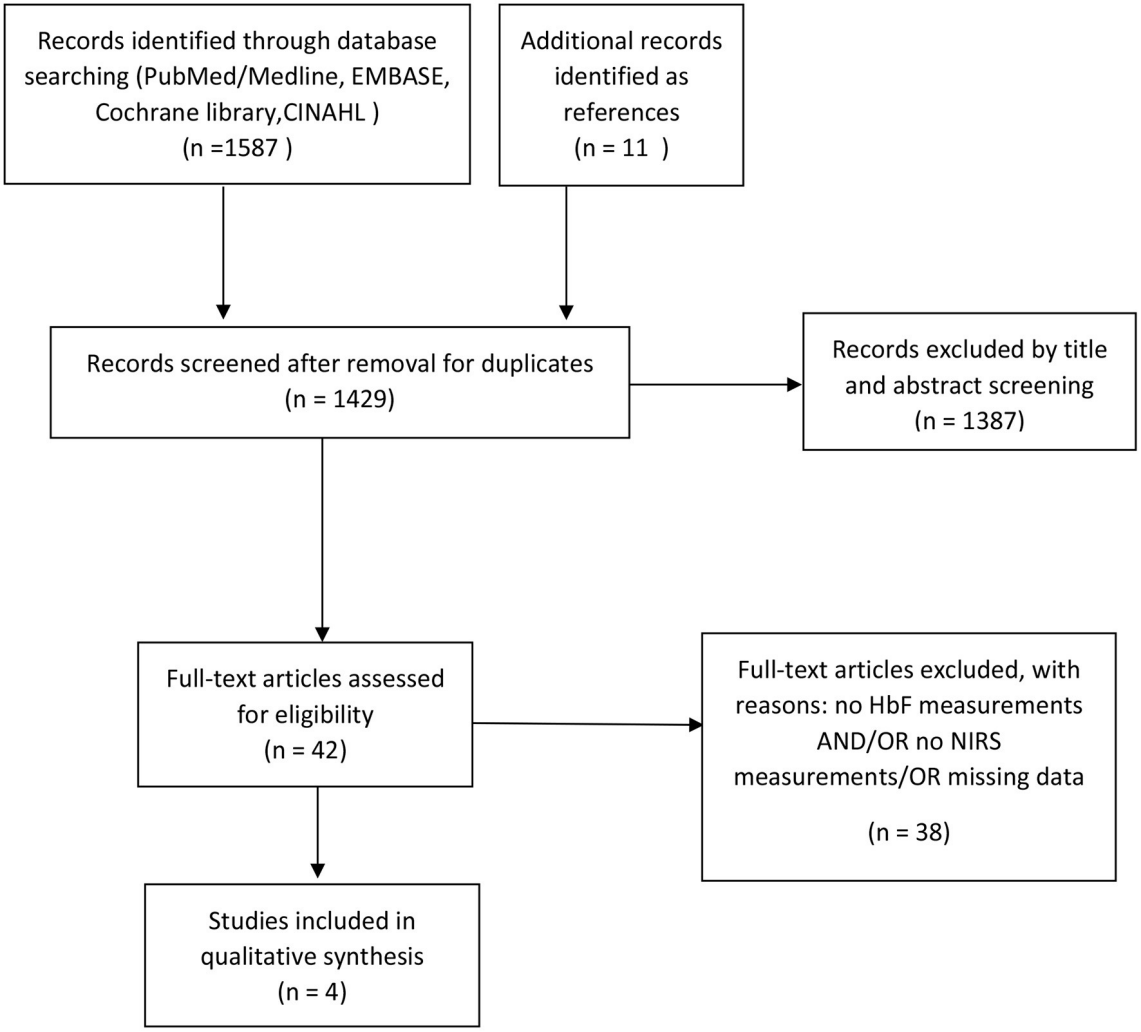
Study selection flow diagram according to the PRISMA statement.

Our initial search identified 1268 articles. After removal of duplicates and rejection (e.g., absence of HbF measurements and/or absence of NIRS measurements in term or preterm neonates) (Figure 2), four observational studies fulfilled our inclusion criteria (Table 1) (28–31). An additional identified study was only available as an abstract and had to be excluded due to missing data (32). All studies performed oxygen saturation monitoring by both pulse oximetry and NIRS in neonates in the first days and/or weeks after birth. The study populations included preterm neonates with a range in gestational age from 25 to 32 weeks. Only two studies reported on the HbF measurement method, which was the absorption spectral analysis by a hemoximeter in both studies (26, 30). All studies used NIRS devices with only one type of probes available and the reported distances between emitter and sensor ranged from 3 to 6 cm for cerebral measurements (28, 30, 31) and 1,5-2,5 cm for peripheral (muscular) measurements (29).

**Table 1:** NIRS Studies with HbF measurements.

**References**	**Number of patients/HbF blood samples**	**Blood sample type**	**HbF measurement method**	**Gestation distribution (weeks)**	**Time of sample collection and non-invasive monitoring**	**Blood gas analyzer/ Hemoximeter**	**Pulse oximeter (company name if available)**	**NIRS device**	**NIRS-derived oxygenation parameters**	**Main results**
Wickrama-singhe et al. (28)	6/NA	Arterial or venous	Visible absorption spectroscopy (hemoximeter)	NA	During 1st week after birth + over 48 days (1 patient)	OSM3 Hemoximeter (Copenhagen, Denmark)	Nellcor N-200 (Tyco Healthcare, Pleasanton, CA, USA)	NA	Cerebral [HbO2]A and [HbO2]F	HbF does not affect Hb absorption coefficients for NIRS
Wardle et al. (29)	96/94	NA	NA	25–32	9–37 days after birth	NA	Datex-Ohmeda, (GE Healthcare, Helsinki, Finland)	NIRO 500 (Hamamatsu Photonics, Japan)	Peripheral FOE (upper forearm)	Positive correlation between FHbF and peripheral FOE
Wardle et al. (30)	91/77	Arterial	NA	26–31	6–31 days after birth + 12–24 h after blood transfusions (for anemic group)	NA	Datex-Ohmeda, (GE Healthcare, Helsinki Finland)	NIRO 500 (Hamamatsu Photonics, Japan)	Cerebral FOE	No correlation between FHbF and cerebral FOE
Naulaers et al. (31)	15/8	Arterial	Visible absorption spectroscopy (hemoximeter)	25–30	First 3 days after birth	Radiometer (Copenhagen, Denmark)	Nellcor 2000	NIRO 300 (Hamamatsu Phototonics, Japan)	Cerebral TOI	No correlation between FHbF and cerebral TOI

*NA, not available; [HbO2]A, cerebral oxyhemoglobin concentration calculated with HbA absorption coefficients; [HbO2]F, cerebral oxyhemoglobin concentration calculated with HbF absorption coefficients; FOE, fractional oxygen extraction; FHbF, fraction of fetal hemoglobin; TOI, tissue oxygenation index*.

Non-invasive SpO2 monitoring was performed in all studies via pulse oximetry at upper or lower extremity. NIRS monitoring included cerebral or peripheral (muscle) measurements. The study by Wickramasinghe et al. investigated whether it was necessary to use the absorption coefficients of HbF, instead of HbA, in the algorithm of NIRS device to calculate the cerebral tissue oxygenation in neonates (28). Two studies performed by Wardle et al. examined whether differences in FHbF in preterm neonates affected peripheral or cerebral fractional oxygen extraction (FOE) (29, 30). The study by Naulaers et al. included FHbF as a factor in a multiple regression analysis of the cerebral tissue oxygenation (31).

Three studies found no influence of FHbF on the NIRS oxygenation parameters (28, 30, 31). In contrast, one study described an effect of FHbF on NIRS measurements, namely a positive correlation between FHbF and FOE (28).

## Discussion

We included four observational studies in the present systematic review on HbF and tissue oxygenation measurements by NIRS. Three cerebral studies did not find HbF to affect the measured NIRS-derived oxygenation parameters whereas one peripheral NIRS study found a positive correlation between FHbF and peripheral FOE.

The study describing a positive correlation between FHbF and peripheral-muscle FOE was the largest (96 patients), designed as a case-control study. It included two groups: symptomatic and asymptomatic anemic preterm neonates receiving transfusions and a control group without a transfusion. Interestingly, there was only a weak correlation between total hemoglobin (Hb) and FOE, but a strong correlation with FHbF and FOE in all of the groups. The measured FOE on upper forearm fell significantly after the transfusion in the symptomatic anemic patients correlating with FHbF decline. These results suggest an improvement in oxygen availability with lower FHbF. The latter was also supported by the fact that the asymptomatic anemic patients had significantly lower initial FHbF compared to the symptomatic patients (21.5 vs. 70%). The difference in initial FHbF was most probably caused by the previous transfusions. The effects of increase in total hemoglobin and red blood cell (RBC) volume on FOE after the transfusion were less significant compared to the effect of FHbF (29).

Three cerebral studies did not find any significant association between FHbF and cerebral oxygenation. The largest cerebral study was a case-control study, which included a transfusion group (low Hb or symptoms of anemia) and a control non-transfusion group. The results showed no effect of different FHbF in both groups on cerebral FOE. A possible explanation for this finding is that the oxygen availability to the brain may be compensated by cerebral autoregulation and consecutive changes in cerebral blood flow, regardless of total Hb and FHbF (30). These results, however, differ from an animal (fetal lamb) model, which showed that the transfusion of adult RBCs results in an increase in cerebral FOE (33). One possible explanation is the greater difference between the oxygen affinity of HbA and HbF in sheep compared to humans (30). An additional explanation is that the rapid changes in FHbF due to the exchange transfusions in fetal lambs *in utero* could not be well-compensated. We can only hypothesize that cerebral autoregulation in human neonates seems to counterbalance the changes in FHbF more efficiently compared to the animal model.

The second smaller cerebral NIRS study investigated whether changes in FHbF affected tissue oxygenation in premature infants during the first 3 days after birth (31). Cerebral tissue oxygenation increased during the 3-day period, but it was unaffected by the changes in FHbF. FHbF values, however, decreased over the 3-day period due to the transfusions of adult RBCs to the neonates. The authors stressed the limitations of the small sample size of only eight premature infants and short NIRS measurement times at each day (30 min). Thus, these results have to be interpreted with caution.

Finally, the last cerebral study tested whether it is necessary to modify the absorption coefficients used in NIRS calculations for the presence of HbF in neonates. Namely, HbA absorption coefficients for the wavelengths used by NIRS differ minimally from those of HbF. The authors found no relevant differences in the calculations, regardless of absorption coefficients used or differences in FHbF of the samples. The main limitations of this study is the sample size of only six patients and the fact that the research question was of a technical and not of a physiological nature (28).

In summary, the main limitation of two of the four included studies is the small sample size (28, 31). Moreover, the two large case-control trials on peripheral (29) and cerebral oxygenation (30) included only preterm neonates at the neonatal intensive care unit, which did not clarify the question of potential HbF affection of FOE in healthy term newborns.

An additional limitation of the two largest studies is the missing information on the measurement methods for HbF, which makes it difficult to assess whether the same method and devices were used, as well as how accurate the HbF measurements were.

Finally, an important clinical aspect regarding FHbF in preterm neonates, is the relationship between FHbF depletion after the transfusion of adult RBCs and oxidative stress related tissue injury. Higher FHbF is already reported to be a protective factor for development of ROP in very preterm infants (8). In the latest published observational study on HbF and bronchopulmonary dysplasia (BPD), rapid postnatal decline in FHbF levels rather than an increased oxygen exposure was associated with development of BPD in very preterm infants (34). A randomized trial addressing a similar question is already recruiting [Preservation of Blood in Extremely Preterm Infants (LIM) ClinicalTrials.gov Identifier: NCT04239690].

Before the gaps in our knowledge concerning the relationship between FHbF and oxidative stress in human neonates are closed, NIRS monitoring of tissue oxygenation, especially after RBC transfusions, could be helpful in understanding this very important physiological question and to clarify the observed differences between the different measured regions (peripheral vs. cerebral).

## Conclusion

Currently available limited data suggest that FHbF could affect peripheral muscle FOE, but seems not to affect cerebral oxygenation in preterm neonates. More studies are needed to draw a final conclusion on this matter, especially concerning the oxygenation changes driven by adult RBC transfusions.

## Data Availability Statement

The original contributions presented in the study are included in the article/supplementary material, further inquiries can be directed to the corresponding author/s.

## Author Contributions

EP and GP conceptualized and designed the review, conducted systematic search of literature, drafted the initial manuscript, and reviewed and edited the manuscript. EP and BS designed the table and reviewed and edited the manuscript. EP, GP, BS, and BU critically reviewed the manuscript for important intellectual content. All authors approved the final manuscript as submitted and agree to be accountable for the content of the work.

## Conflict of Interest

The authors declare that the research was conducted in the absence of any commercial or financial relationships that could be construed as a potential conflict of interest.

## Publisher's Note

All claims expressed in this article are solely those of the authors and do not necessarily represent those of their affiliated organizations, or those of the publisher, the editors and the reviewers. Any product that may be evaluated in this article, or claim that may be made by its manufacturer, is not guaranteed or endorsed by the publisher.
